# Sexual and reproductive health policies for migrant, immigrant and refugee populations in select high-income countries: a policy analysis protocol

**DOI:** 10.1136/bmjopen-2025-107994

**Published:** 2025-12-10

**Authors:** Negin Mirzaei Damabi, Patience Castleton, Zelalem Mengesha, Zachary Munn, Mumtaz Begum, Jodie Avery, Salima Meherali, Zohra Lassi

**Affiliations:** 1 The University of Adelaide School of Public Health, Adelaide, South Australia, Australia; 2 University of Canberra, Canberra, Australian Capital Territory, Australia; 3 JBI, The University of Adelaide Faculty of Health and Medical Sciences, North Adelaide, South Australia, Australia; 4 Adelaide Medical School, The University of Adelaide Faculty of Health and Medical Sciences, Adelaide, South Australia, Australia; 5 PROS, Medicine, The University of Adelaide, Adelaide, South Australia, Australia; 6 Faculty of Nursing, University of Alberta, Edmonton, Alberta, Canada; 7 School of Public Health, The University of Adelaide School of Public Health, Adelaide, South Australia, Australia

**Keywords:** Refugees, Health Equity, Public health, Health policy, SEXUAL MEDICINE

## Abstract

**Abstract:**

**Introduction:**

International migrants comprise 3.6% of the global population and face systemic barriers to accessing sexual and reproductive health (SRH) services, such as contraception, safe abortion care and sexual function support. In high-income countries, policy frameworks vary widely, with migration status significantly influencing entitlement and access to host countries. This protocol outlines a planned study to systematically analyse SRH policies in high-income countries with strong migrant integration frameworks, aiming to identify policy gaps, assess inclusivity and inform recommendations to strengthen Australia’s SRH policy landscape.

**Methods and analysis:**

This study employs a systematic policy analysis using the Joanna Briggs Institute scoping review methodology. Countries with ≥10% migrant populations and a Migrant Integration Policy Index health score ≥70 will be included. 13 countries meet these criteria, including Australia, Canada and Sweden. A comprehensive search of academic databases (PubMed, Scopus, Web of Science, Cumulative Index to Nursing and Allied Health Literature and ProQuest Public Health) and grey literature from governmental and non-governmental sources will be conducted. Data extraction will follow Bacchi’s ‘What’s the Problem Represented to Be?’ approach. Thematic analysis will combine deductive and inductive methods to examine the extent to which SRH policies address migrant and refugee needs, including sexual function, safe abortion care and fertility care. A comparative policy matrix will identify strengths, limitations and best practices.

**Ethics and dissemination:**

As this study analyses publicly available policy documents, ethics approval is not required. Findings will be disseminated through peer-reviewed publications and policy briefs targeting stakeholders involved in SRH policy and migrant health.

**Registration details:**

This protocol is registered with the Open Science Framework (OSF): https://doi.org/10.17605/OSF.IO/AYZ6P

**STRENGTHS AND LIMITATIONS OF THIS STUDY**

- The use of Bacchi’s ‘What’s the Problem Represented to Be?’ framework enables a critical, theory-informed analysis of how SRH policies construct and address migrant and refugee needs.
- Inclusion of both academic and grey literature sources ensures comprehensive coverage of policy documents from government and non-governmental organisations.
- The inclusion criteria—based on the Migrant Integration Policy Index health scores and migrant population thresholds—ensure relevance but may exclude countries with emerging but less documented policy efforts.
- This study centres on only high-income countries; therefore, no countries from Asia, Africa, Central or South America or the Caribbean are included, which may limit the relevance of outcomes to a global audience.
- Authors only have the capacity to include documents published in the English language, thus further limiting the included countries to those whose primary language is English.

## Background

 Migration is a defining feature of the modern global landscape, with international migrants comprising 3.7% of the world’s population in 2023.^1^ At the conclusion of 2023, 117.3 million individuals had been forcibly displaced, including 43.3 million refugees.^2^ This growing demographic faces unique challenges in accessing essential services, particularly sexual and reproductive health (SRH) care.^3^ SRH encompasses critical areas such as sexual function and psychosexual counselling, safe abortion care, fertility care, gender-based violence and prevention of HIV and sexually transmitted infections (STIs).^4^ However, systemic barriers, including restrictive policies, legal uncertainties, financial constraints and cultural differences, often hinder access to these services, resulting in disproportionately poor SRH outcomes for migrant and refugee populations.^5^

Globally, the importance of equitable access to SRH services is underscored by international frameworks such as the United Nations Sustainable Development Goals (SDGs). Specifically, Goal 5, where the need to achieve gender equality and empower all women and girls has been promoted, and Goal 10, which emphasises reducing inequalities within and among countries.^6^ In Australia, national strategies further highlight the urgency of addressing these disparities. The National Women’s Health Strategy 2020–2030 identifies maternal SRH as a priority area for all women and underscores health equity between women as a key policy focus.^7^ Similarly, SRH is identified as a priority area of intervention in the Australian National Men’s Health Strategy 2020–2030, which also emphasises health equity among different groups of men, including those from culturally and linguistically diverse backgrounds, migrants and asylum seekers.^8 9^ Both strategies highlight the need to address gaps in services for vulnerable groups that are at higher risk of experiencing worse health outcomes.

Despite these commitments, significant disparities persist in SRH outcomes for migrants and refugees compared with non-migrant populations. For example, migrant populations in Australia experience higher rates of STIs and HIV due to limited access to preventive healthcare services.^10 11^ Additionally, studies reveal that over 65% of migrants in some high-income countries have inadequate health literacy, which negatively affects their ability to access preventive healthcare and SRH services. While these substantial disparities in health outcomes exist, it is important to understand the public policies and guidelines that prohibit and facilitate access to culturally sensitive and appropriate health information and services, particularly SRH. In the context of Australia, public policies are developed by local and national governmental bodies, organisations and agencies to guide resource and funding allocations, government-led initiatives and laws. Public policy is guided by research and previous policies and implemented by national or local governing bodies.^12^ Health policy in Australia helps to guide SRH protection and rights for all people. For example, the 2020–2030 National Women’s Health Strategy prioritises increasing access to SRH information and services for diverse communities around the country.^13^

The complex and diverse policies and legislations that exist in each country strongly govern the ways that men and women from migrant and refugee backgrounds understand and care for their SRH. In many high-income countries, including Australia and the UK, access to standard healthcare is granted to all legal migrants. However, a country’s policy, guidelines and recommendations often differ depending on an individual’s migration status, such as migrant, refugee or asylum seeker status or residency status (temporary or permanent resident), creating a confusing web of information to navigate on arrival to a new country. Refugees and asylum seekers are particularly vulnerable, frequently excluded from publicly funded healthcare services or subjected to additional costs and complex referral processes. For example, prenatal and postpartum care in the USA often involves significant out-of-pocket expenses for undocumented migrants.^14 15^ However, diversity exists in SRH and migrant policies around the world, with many countries implementing specific policies to protect their SRH, including mandatory HIV testing for refugees entering New Zealand.^16^ Nonetheless, previous research highlights persistent global policy gaps in areas such as men’s SRH and women’s sexual function.^17^

Additionally, there is a large gap in general health-related policies and practices for migrants, despite the evidence that shows their need for specialised and sensitive care that protects their unique backgrounds and migration experiences. This protocol aims to examine whether and how international and national research policies in high-income countries address these domains related to migrant and refugee populations. Analysing existing SRH policies in countries with strong migrant integration frameworks is essential to identifying best practices and policy gaps to inform policy recommendations in Australia, supporting equitable access to SRH services regardless of migration status. With Australia as the primary focus, recommendations will be directed towards strengthening its SRH policy landscape to improve attempts at including and acknowledging the needs of migrants and refugees. Analysing other high-income countries' policies ensures that these recommendations are evidence-based, globally benchmarked and informed by diverse approaches to migrant SRH policy.

Additionally, there is a large gap in general health-related policies and practices for migrants, despite the evidence that shows their need for specialised and sensitive care that protects their unique backgrounds and migration experiences. This protocol aims to examine whether and how international and national research policies in high-income countries address these domains related to migrant and refugee populations. Analysing existing SRH policies in countries with strong migrant integration frameworks is essential to identifying best practices and policy gaps to inform policy recommendations in Australia, supporting equitable access to SRH services regardless of migration status. With Australia as the primary focus, recommendations will be directed towards strengthening its SRH policy landscape to improve attempts at including and acknowledging the needs of migrants and refugees. Analysing other high-income countries’ policies ensures that these recommendations are evidence-based, globally benchmarked and informed by diverse approaches to migrant SRH policy.

This paper provides a detailed outline of the protocol for our policy analysis exercise that aims to understand the best practices that shape SRH access for migrant and refugee populations.

### Research questions

- What national-level SRH policies currently exist in countries with strong migrant integration frameworks (Migrant Integration Policy Index (MIPEX) health score ≥70, slightly favourable to favourable policies and ≥10% migrant population)?
- To what extent do these SRH policies attempt to meet the needs of migrant and refugee populations, including undocumented migrants and asylum seekers?
- Which SRH domains, based on the WHO framework^18^ (eg, safe abortion care, fertility care, gender-based violence and sexual function and psychosexual counselling), are covered in these policies, and what gaps remain?
- How do selected countries incorporate principles of equity, cultural responsiveness and rights-based approaches in the design and implementation of SRH policies for migrants and refugees?
- What are the key policy innovations, best practices and barriers identified across countries, and how can these inform improvements to Australia’s SRH policy landscape?

## Methods and analysis

### Study design and theoretical framework

This policy/document analysis adopts a scoping methodology guided by the Joanna Briggs Institute framework for scoping reviews, which provides structured guidance for question formulation, study selection, data extraction and synthesis.^19 20^ To ensure transparency, consistency and comprehensiveness in reporting, the Preferred Reporting Items for Systematic Reviews and Meta-Analyses extension for Scoping Reviews (PRISMA-ScR) checklist will be followed.^21^ The protocol has been registered with the Open Science Framework (OSF) to promote methodological transparency and replicability (https://doi.org/10.17605/OSF.IO/AYZ6P). The timeline for this review is expected to be between November 2025 and February 2026.

### Eligibility criteria

Eligibility criteria are structured according to the Population–Concept–Context framework, which is appropriate for scoping reviews aimed at the analysis of evidence across diverse policy settings.^20^

### Population

Migrant and refugee populations. We will include men and women and gender-diverse individuals across the life course, with a particular emphasis on policies that reference or apply to reproductive-aged individuals (15–49 years), where applicable. The review focuses on policy documents that address the needs of international migrants, including refugees, asylum seekers and, where relevant, undocumented migrants. To ensure clarity, definitions of key terms such as migrant, immigrant, refugee, asylum seeker and undocumented migrant are provided in table 1.

**Table 1:** Definitions of the included population groups

Term	Definition
Migrant	Any person who moves across an international border away from their country of usual residence, regardless of legal status, cause of movement or length of stay.^34^
Immigrant	A person who moves into a country other than that of their nationality or usual residence, usually for long-term settlement.^35^
Refugee	A person forced to flee their country due to persecution, conflict or violence and recognised under international law (1951 Refugee Convention).^34^
Asylum seeker	An individual seeking international protection whose claim for refugee status has not yet been determined.^36^
Undocumented migrant	A person who enters, stays or works in a country without the necessary authorisation or valid documents required under immigration regulations.^37^

The inclusion of comparator countries enables systematic benchmarking, ensuring that policy recommendations for Australia are informed by an understanding of global innovations, strengths and limitations in SRH policy of other high-income countries***.*** The selection of countries and documents reflects two inclusion groups:

### Group 1: national policies from selected high-income countries

To ensure both demographic significance and policy maturity in addressing migrant health, countries were selected based on two criteria:

- Migrant population proportion: countries where migrants constitute at least 10% of the total population will be included in the review. This criterion ensures the inclusion of countries with a significant migrant presence, and thus policies are more likely to address migrant and refugee health needs. Data on migrant population percentages were obtained from the United Nations (2020) International Migration Dataset, which provides country-level statistics on the proportion of foreign-born residents.^22^
- MIPEX health score: English-speaking countries with a MIPEX health score of 60 or higher will be included in the review, as these are countries with slightly favourable to favourable policies.^23^ The MIPEX is a widely recognised comparative tool that assesses national policies across four key health dimensions: entitlement to health services, accessibility of healthcare, responsiveness of health services and measures to achieve change.^23^ Countries are scored on a scale from 0 to 100, with higher scores indicating stronger policies that support equitable healthcare access for migrants. Only English-speaking countries were chosen due to the limited translation capacity of complex policy documentation.^23^
- MIPEX data were retrieved from the 2020 MIPEX Report, which assesses 56 countries, including all EU member states, the UK, the USA, Canada and Australia.^24^

These criteria allow for the inclusion of countries where the intersection of SRH policies and migrant health is both relevant and measurable.

The final list of countries to be included in this review is presented in table 2.

**Table 2:** Selected countries with MIPEX health scores and migrant population percentages

Countries	MIPEX health score	Immigrants (n)	% Population
Ireland	85	871.3K	17.64%
New Zealand	83	1.4M	28.65%
USA	79	50.6M	15.28%
Australia	79	7.7M	30.14%
UK	75	9.4M	13.79%
Canada	73	8M	21.33%

K, thousand; M, million; MIPEX, Migrant Integration Policy Index.

### Group 2: global and regional policy frameworks

Documents with a global scope will be included when they:

- Are issued or endorsed by international or intergovernmental organisations (eg, WHO, United Nations Population Fund (UNFPA) and International Organization for Migration (IOM)).
- Provide high-level guidance relevant to SRH service provision for migrant and refugee populations.

Exclusion: documents that focus on low/middle-income countries (LMICs) will be excluded from this review, regardless of their MIPEX health score or migrant population proportion. This is due to the differences in policy and healthcare system capacities that LMICs have in comparison to high-income countries. As LMICs are often faced with constraints of resources and infrastructure to support SRH services,^25^ resulting in resource allocation mainly focusing on immediate public health concerns, such as communicable diseases and children’s health.^26^ Additionally, much of the health economics research, especially with migrants and refugees, in LMICs is driven by external agencies, such as the UN and the WHO, whose policies then often reflect trends in HICs.^27^ As a primary aim of this research is to guide Australian SRH policy, the review will include only policies from other HICs that offer contextual relevance.

### Concept: SRH policy domains

To be included, documents must address at least one of the SRH domains defined by the WHO framework for operating SRH.^18^ These domains and their definitions are outlined in table 3.

**Table 3:** Content selection domains for inclusion

SRH domain	Definition/scope	Inclusion relevance
Prevention and control of HIV and other STIs	Prevention, screening, testing, treatment and counselling related to sexually transmitted infections and HIV/AIDS; includes access to condoms and assisted reproductive technologies (ARTs).	Includes policies on STI prevention and care, especially those with provisions for vulnerable populations, including those identifying as Lesbian, Gay, Bisexual, Transgender, Intersex, Queer, plus other identities, like Asexual (LGBTIQ+).
Contraception counselling and provision and family planning	Access to contraceptive methods, family planning services and counselling, including reproductive choice education.	Policies promoting all forms of contraceptive access and/or outlining service provision models.
Antenatal, intrapartum, postnatal, maternal and perinatal health	Antenatal, childbirth and postnatal care to support maternal and newborn health; includes skilled birth attendance.	Relevant where maternal health programmes include equity goals or migrant-sensitive strategies.
Safe abortion and postabortion care	Safe and legal abortion services, postabortion care and related support services, including mental health support.	Includes documents that address abortion access and laws surrounding it or outline service pathways and counselling.
Gender-based violence (GBV) prevention, support and care	Prevention and response to GBV, including sexual violence and intimate partner violence; provision of survivor services, mental health support and legal protections.	Policies addressing GBV response mechanisms, especially if inclusive of vulnerable or at-risk groups.
Adolescent SRH	Services and education tailored to adolescents, including topics on consent, puberty, contraception and healthy relationships.	Includes national education strategies or youth health policies referencing adolescent SRH.
Reproductive cancers	Prevention, screening, early detection and treatment of cancers affecting reproductive systems (eg, cervical, breast, prostate).	Policies with national screening programmes or vaccination initiatives (eg, HPV) may be relevant.
Comprehensive education and information on sexual health and well-being	Promotion of positive, respectful sexuality and safe sexual relationships; includes comprehensive sexuality education and rights-based frameworks.	Policies recognising sexual rights, gender identity or inclusion in sexual education may be included.
Sexual function and psychosexual counselling	Identification, prevention and treatment of sexual concerns and difficulties, including sexual dysfunctions and disorders. Providing supportive and specific information and advice in a culturally appropriate manner.	Policies with national treatment plans for sexual dysfunctions that recognise equal opportunities for support and prevention.
Fertility care	Prevention and screening of infertility for men and women and the provision of assisted fertility care for all.	Includes access to assisted reproductive technologies, such as in-vitro fertilisation and strategies to raise awareness of fertility struggles.

SRH, sexual and reproductive health; STI, sexually transmitted infection.

### Context: policy landscape and document types

This review will include documents that represent national and international-level policy/guideline recommendations from government and non-governmental bodies. Eligible documents must be officially issued, endorsed or adopted by national governments or authorised public institutions, such as ministries of health, intersectoral agencies, equivalent national bodies or intergovernmental organisations. The inclusion covers both stand-alone SRH policy frameworks and broader health strategies that encompass SRH and migrant/refugee components.

Relevant documents may include national SRH policies, health sector strategic plans, implementation frameworks, technical guidelines and protocols. National action plans on specific SRH domains, such as those highlighted in table 3, are also within the scope of inclusion, provided they are endorsed or adopted by a national-level authority. Prevention and disease management-related policies will be eligible for inclusion. Additionally, white papers, official public health reports with policy implications and international or regional frameworks that have been formally integrated into policy frameworks will be considered eligible.

To facilitate clarity and consistency during the selection process, the following definitions will be used to distinguish document types included in the review. Table 4 outlines the operational definitions and examples used to classify included documents:

**Table 4:** Document typology with definitions and examples

Document type	Definition	Example
Policy	The intentional process in which an unforeseeable number of actors, bearers of specific interests and ideas, continuously interact with each other in order not only to maintain or increase their power but also to deal with and to solve problems perceived to have collective relevance or impact.^38^	Child Fund Australia’s Sexual and Reproductive Health Policy.^39^
Technical report/practical guideline	A technical or practical guideline is a systematically developed document that provides evidence-based recommendations to guide decision-making in specific areas of practice. These guidelines are designed to assist professionals, policymakers, and stakeholders in making informed choices about interventions, procedures, or policies to achieve optimal outcomes.^40^	The revised international technical guidance on sexuality education—a powerful tool at an important crossroads for sexuality education.^41^
Implementation framework	An implementation framework is a structured approach used to guide the systematic adoption, integration, and sustainability of policies, programmes, or interventions within specific settings. Rooted in implementation science, these frameworks help bridge the gap between evidence-based recommendations and real-world practice by identifying key factors that influence successful implementation.^42^	Intervention Framework, Sexual and Reproductive Health and Rights.^43^
National strategy/national plan	Detailed document, usually developed by governmental organisations, that outlines the ways in which they will address a specific issue in order to achieve a national goal.^44^	National Hepatitis B Strategy, Australia.^45^
Roundtable recommendations	Roundtable recommendations are documents that emerge from roundtable meetings in which field experts discuss emerging trends and issues related to a specific health issue or concern. The recommendations drive policy and guide decision-making.^46^	Syphilis roundtable, 22 October 2021— Ministerial Advisory Committee on Blood Borne Viruses and Sexually Transmissible Infections (MACBBVSTI) recommendations.^47^

These classifications will be applied during screening and data extraction to ensure consistent categorisation of included documents and transparency in methodological reporting.

While empirical studies, policy evaluations and systematic reviews may be encountered during the literature search, they will not be included in the analysis per se. Instead, the review team will systematically examine the reference lists of such studies to identify and retrieve original policy documents. Only those primary documents that meet the predefined inclusion criteria will be included in the review. All included documents must be published between 2015 and 2025, ensuring that the analysis captures contemporary policy developments aligned with the SDGs’ era. If the same document has been updated multiple times between 2015 and 2025, only the latest document update will be eligible for inclusion. Only documents published in the English language will be accepted.

### Data sources and search strategy

The search will be conducted across a combination of academic databases and policy/grey literature sources to ensure a comprehensive identification of relevant SRH policies for migrant and refugee populations.

The academic database search will include PubMed, Scopus, Web of Science, ProQuest Public Health and Cumulative Index to Nursing and Allied Health Literature (CINAHL). However, these articles will not be included in the final screening. Instead, their reference lists will be systematically examined to identify original SRH policy documents. Only these primary documents, if meeting the eligibility criteria, will be included in the review.

In addition to academic sources, the review will incorporate policy and grey literature sources, which are crucial for capturing government and non-government policy documents, reports and guidelines. These sources will include government websites of the selected countries, as national policies and legislative documents are often published on official government portals. The websites of international organisations and agencies such as the WHO, the IOM and the UNFPA will be included, as they publish critical global and regional reports on migrant health policies. Reports from international non-governmental organisations, including Médecins Sans Frontières, Save the Children and the Multicultural Centre for Women’s Health, will also be included in our web-based search, as these organisations frequently assess healthcare access and policy implementation for migrants and refugees.

Additionally, searches within key institutions with significant contributions to policy evaluations and migrant health research, including Organisation for Economic Co-operation and Development, Cochrane Public Health Group, World Bank Health Policy Reports, Guttmacher Institute and The Lancet Migration Initiative, will be conducted. National health department websites from the selected countries will also be reviewed for country-specific SRH policies, guidelines and implementation reports.

To ensure a systematic and replicable search, we developed a structured search strategy in consultation with a qualified health sciences librarian. The strategy used Medical Subject Headings (MeSH) terms and relevant keywords across three primary domains: population (migrants, refugees), SRH and policy. The search will employ Boolean operators to combine terms, ensuring precision and comprehensiveness. Keywords such as “Migrant” OR “Refugee” OR “Asylum Seeker” OR “Culturally and Linguistically Diverse” will be combined with “Sexual Health” OR “Reproductive Health” OR “Family Planning” OR “HIV Treatment” and “Policy” OR “Policies” OR “Legislation” OR “Framework” OR “Guidelines”. The search will be restricted to the selected countries that meet the eligibility criteria to maintain relevance to the study’s objectives. The detailed search strategy is provided as an online supplemental file 1.

This multisource search strategy will enable a comprehensive analysis of existing SRH policies for migrant and refugee populations, ensuring that both academic evidence and policy frameworks are analysed to provide a thorough policy comparison and recommendations.

Web-based grey literature searches and searches within the above-mentioned key agencies will be conducted by hand using a consolidated, structured search strategy that follows the same structure as that of the one developed using MeSH terms. A more refined, simpler keyword search of grey literature will ensure that all relevant articles are screened for eligibility. Keywords such as “Migrant” OR “Refugee” OR “Asylum Seeker” OR “Culturally and Linguistically Diverse” and “Sexual Health” OR “Reproductive Health” OR “Family Planning” OR “HIV Treatment” and “Policy” OR “Policies” OR “Legislation” OR “Framework” OR “Guidelines” will be combined in multiple ways for simple 3- to 5-word searches of the grey literature. This will ensure that all relevant documents are captured and assessed for their relevance.

### Policy selection and screening

The selection process will follow a systematic and transparent approach to ensure the relevance and quality of the included documents. For records retrieved from academic databases (eg, PubMed, Scopus, CINAHL, ProQuest Public Health and Web of Science), screening and review will be conducted using Covidence, a web-based tool designed to streamline the selection process, manage reviewer workflows and ensure auditability.^28^ Two independent reviewers will conduct title and abstract screening within Covidence to assess whether documents meet the inclusion criteria. Eligible records will proceed to a full-text review, where inclusion will be confirmed based on predefined eligibility criteria.

For grey literature and policy documents retrieved from government and organisational websites, screening will be conducted manually. Documents and relevant web pages will be imported into a standardised Excel spreadsheet. Two independent reviewers will screen each entry, applying the same inclusion criteria used for academic records.

To ensure consistency and inter-reviewer reliability across both types of sources, a pilot screening phase will be undertaken prior to the main screening process. This calibration exercise will involve a small sample of academic and grey literature documents, enabling the review team to align on inclusion criteria, resolve ambiguities and refine the screening tools and procedures as necessary.^20^ Discrepancies at any stage will be resolved through discussion and, if needed, by involving a third reviewer.

The entire document selection process will be documented using a PRISMA-ScR flow diagram, reporting the number of records identified, screened, reviewed, excluded (with reasons) and included in the final synthesis.

Following the full-text screening, a structured data extraction process will be undertaken to systematically chart relevant information from the included policy documents. A standardised data extraction form (online supplemental file 2), developed and piloted by the review team, will guide the process to ensure clarity, consistency and replicability across reviewers. Any discrepancies will be resolved through discussion and, if needed, consultation with a third reviewer.

The extraction framework is grounded in the six questions developed in Bacchies’ ‘What the Problem Represented to Be?’ (WPR) approach.^29^ Following this framework allows for critical analysis of the problems, assumptions and implications of the policy. This framework was selected for its ability to question what the actual equitable care provision is being offered in the policy, allowing for analysis of how the SRH of refugees and migrants is being framed or missed in the documents. The extraction form will be piloted and refined as needed to ensure clarity and consistency across reviewers.

The form will capture, but will not be limited to, the following categories. These guiding questions are presented in box 1.

Box 1 **WPR guiding questions**

**Question 1:** what’s the problem (eg, of ‘gender inequality’, ‘drug use/abuse’, ‘economic development’, ‘global warming’, ‘childhood obesity’, ‘irregular migration’, etc) represented to be in a specific policy or policies?

**Question 2:** what deep-seated presuppositions or assumptions (conceptual logics) underlie this representation of the ‘problem’ (problem representation)?

**Question 3:** how has this representation of the ‘problem’ come about?

**Question 4:** what is left unproblematic in this problem representation? Where are the silences? Can the ‘problem’ be conceptualised differently?

**Question 5:** what effects (discursive, subjectification, lived) are produced by this representation of the ‘problem’?

**Question 6:** how and where has this representation of the ‘problem’ been produced, disseminated and defended? How has it been, and/or how can it be disrupted and replaced?

Questions adopted from Bacchies ‘What the Problem Represented to Be?’ (WPR) approach.^29^

### Data synthesis and analysis

The analysis will employ an integrated descriptive and thematic synthesis approach^30^ guided by the WPR policy analysis model; the synthesis aims to offer a structured, comparative and policy-relevant overview of how national SRH policies address access for migrant and refugee populations.

### Descriptive synthesis

A descriptive analytical framework will be used to summarise and visualise key characteristics of the included policy documents. This will involve:

- Policy landscape analysis: tabulation of documents by country, year, type (eg, strategy, law, guideline), issuing authority and publication language.
- Inclusion metrics: quantification of documents that explicitly reference migrants, refugees, asylum seekers or undocumented populations, including subgroup distinctions by gender, age or status.
- Access dimension profiling: analysis of policy content across the WPR model, using binary and categorical indicators to assess presence/absence and depth of access considerations.
- SRH domain coverage: assessment of coverage across WHO-defined SRH domains (eg, contraception, maternal health, gender-based violence, HIV/STIs, adolescent SRH).
- Scope and reach: documentation of governance levels (national, regional and local), integration into mainstream health systems and inclusion in emergency/humanitarian settings.

Descriptive results will be presented in tables and visualised using charts to facilitate comparison across countries and domains.

### Thematic analysis

The thematic synthesis will adopt both deductive and inductive approaches,^31^ drawing from the prestructured data extraction framework grounded in the WPR model. NVivo or similar qualitative analysis software will be used to support systematic coding and inter-reviewer consistency.

### Comparative and cross-country synthesis

A comparative dimension will identify both shared approaches and divergences in policy design and implementation:

- Cross-national patterns: comparison of inclusion practices across different governance and health system contexts.
- Exemplary practices: highlighting policies that demonstrate comprehensive migrant inclusion, high cultural responsiveness or strong integration mechanisms.
- Structural barriers: identifying contradictions or gaps—such as eligibility exclusions or absence of migrants and refugee population—that hinder equitable SRH access.

This comparative lens will help assess the extent to which countries with high migrant representation align with principles of universality, equity and cultural responsiveness in their SRH policy frameworks.

### Ethics and dissemination

As this study involves the analysis of publicly available policy documents and does not involve human subjects, ethical approval is not required. The findings from this review will be disseminated through multiple channels to ensure broad reach and impact. Results will be published in peer-reviewed academic journals.

To ensure comprehensive and nuanced analysis, this review will adopt a gender-disaggregated approach to policy examination and reporting. This decision is informed by international frameworks that emphasise the importance of sex-disaggregated data and gender-specific health strategies. The WHO Global Strategy for Women’s, Children’s and Adolescents’ Health (2016–2030) highlights the need for gender-responsive health policies that address the distinct biological, social and cultural factors affecting health outcomes.^32^ Similarly, the Australian National Men’s Health Strategy (2020–2030) emphasises the importance of sex disaggregation in data and research, enabling the development of gender-specific intervention and policy approaches to ensure that no groups are overlooked and gender-specific barriers are adequately addressed.^33^ SRH policies are inherently gendered, with distinct policy priorities, service delivery models and access barriers affecting women and men differently. Women’s SRH policies typically emphasise maternal health, contraception and gender-based violence, while men’s SRH policies often focus on sexual function, prostate health and reproductive counselling. These differences reflect not only biological variations but also social constructions of gender, gendered help-seeking behaviours and systemic inequalities in healthcare provision. The complexity and distinct nature of these gendered policy issues warrant separate, in-depth analysis to avoid oversimplification and to ensure that gender-specific priorities and gaps are adequately identified.

Therefore, following the completion of data extraction and analysis using the unified methodology outlined in this protocol, findings will be reported in two complementary manuscripts: one focused specifically on SRH policies for women and the other on SRH policies for men. Both manuscripts will employ the same methodology, search strategy and data extraction framework, ensuring methodological rigour and enabling direct comparison of policy approaches, priorities and gaps across gender categories. This parallel analysis will allow for nuanced interpretation of gender-specific policy contexts while maintaining consistency in analytical approach, thereby supporting evidence-based recommendations that address the unique health needs and systemic inequalities affecting migrant and refugee populations across all genders.

### Patient and public involvement

Patients or the public were not involved in the design, conduct, reporting or dissemination plans of this research.

## Supplementary material

10.1136/bmjopen-2025-107994 online supplemental file 1

10.1136/bmjopen-2025-107994 online supplemental file 2
