# Supplementary material for: Sexual and reproductive health policies for migrant, immigrant and refugee populations in select high-income countries: a policy analysis protocol
Source: BMJ Open. 2025 Dec 10;15(12):e107994. doi: 10.1136/bmjopen-2025-107994 (PMC12699594; doi:10.1136/bmjopen-2025-107994)
Supplement: online supplemental file 1 [file bmjopen-15-12-s001.docx]

**Supplementary file 1**

**PubMed**

**Date of Search:** 22 April 2025 **Search Filters Applied:** (2015–2025), Books and Documents, Government Publications, Guidelines, Legal Cases, Legislation, Practice Guidelines, and Reviews. The search also included various categories of research support: American Recovery and Reinvestment Act, NIH (Extramural), NIH (Intramural), Non-U.S. Government, U.S. Government (Non-P.H.S.), U.S. Government (P.H.S.), and general U.S. Government support. Scientific Integrity Reviews were also included.

**Number of Results:** 457

"Transients and Migrants"[mh] OR "Refugees"[mh] OR "Refugee Camps"[mh] OR "Ethnic Groups"[mh] OR "Culturally and Linguistically Diverse"[tiab] OR CALD[tiab] OR Non-English speaking[tiab] OR Migrant worker*[tiab] OR International student*[tiab] OR Labor migrant*[tiab] OR Minority group*[tiab] OR Asylum seeker*[tiab] OR Displaced people[tiab] OR Immigrant*[tiab] OR Undocumented migrant*[tiab] OR Illegal immigrant*[tiab] OR Stateless person*[tiab] OR Climate refugee*[tiab] OR Internally Displaced Person*[tiab] OR IDP[tiab] OR Economic migrant*[tiab] OR Temporary migrant*[tiab] OR Human trafficking victim*[tiab] OR Unaccompanied minor*[tiab] OR Migrant sex worker*[tiab] OR Female migrant*[tiab] OR Male migrant*[tiab] OR Migrant wom*n[tiab] OR Migrant men[tiab] OR LGBTQI+[tiab] migrant*[tiab] OR Sexual minority migrant*[tiab] OR Elderly migrant*[tiab] OR Aging migrant*[tiab] OR Migrant famil*[tiab] OR Limited English proficiency[tiab] OR Newly arrived migrant*[tiab] OR Cross-border migrant*[tiab] OR Expatriate*[tiab] OR Seasonal migrant*[tiab] OR Guest worker*[tiab] OR Migrant healthcare worker*[tiab]

AND

"Sexual Health"[mh] OR "Reproductive Health"[mh] OR "Family Planning Services"[mh] OR "Contraception"[mh] OR "Abortion, Induced"[mh] OR "Sexually Transmitted Diseases"[mh] OR "Sexually Transmitted Diseases, Bacterial"[mh] OR "Sexually Transmitted Diseases, Viral"[mh] OR "HIV Infections"[mh] OR "Acquired Immunodeficiency Syndrome"[mh] OR "Gender-Based Violence"[mh] OR "Sexual Dysfunctions, Psychological"[mh] OR "Sexual Behavior"[mh] OR "Sex Education"[mh] OR "Sexual and Gender Minorities"[mh] OR "Intimate Partner Violence"[mh] OR Trauma-Informed Care*[tiab] OR Unintended Pregnan*[tiab] OR Postabortion Care*[tiab] OR Emergency Contraception*[tiab] OR Sexual Rights*[tiab] OR Reproductive Rights*[tiab] OR Sexual health*[tiab] OR Sexual dysfunction*[tiab] OR Sexual arousal*[tiab] OR Libido*[tiab] OR Orgasm*[tiab] OR Sexual pain*[tiab] OR Sexual satisfaction*[tiab] OR Intimacy*[tiab] OR Sexuality*[tiab] OR Sexual violence*[tiab] OR Sexual abuse*[tiab] OR Rape*[tiab] OR Forced marriage*[tiab] OR Human trafficking*[tiab] OR Female genital mutilation*[tiab] OR Sexual exploitation*[tiab] OR Sexual harassment*[tiab] OR Gender-based violence*[tiab] OR Survivors of sexual violence*[tiab] OR Reproductive coercion*[tiab] OR Sexual consent*[tiab] OR Sexual autonomy*[tiab] OR Sexual health education*[tiab] OR Sexual and reproductive rights*[tiab] OR Sexual well-being*[tiab] OR Stigma and sexuality*[tiab] OR Pleasure-centered sexual health*[tiab] OR Migrant sexual health*[tiab] OR Refugee reproductive health*[tiab] OR LGBTQ+ sexual health*[tiab] OR Culturally sensitive sexual health services*[tiab] OR Sexual dysfunction treatment*[tiab] OR Mental health and sexual health*[tiab]

AND

"Health Policy"[mh] OR "Public Policy"[mh] OR "Guidelines as Topic"[mh] OR "Legislation, Medical"[mh] OR Polic*[tiab] OR Policie*[tiab] OR "SRH Polic*"[tiab] OR Framework*[tiab] OR Guideline*[tiab] OR Legislation*[tiab] OR Law*[tiab] OR Strateg*[tiab] OR "National strateg*"[tiab]

**Scopus**

**Date of Search:** 22 April 2025 **Search Filters Applied:** 2015–2025

**Number of Results:** 1404

TITLE-ABS-KEY("transients and migrants" OR refugees OR "refugee camps" OR "ethnic groups" OR "culturally and linguistically diverse" OR CALD OR "non-english speaking" OR "migrant worker*" OR "international student*" OR "labor migrant*" OR "minority group*" OR "asylum seeker*" OR "displaced people" OR immigrant* OR "undocumented migrant*" OR "illegal immigrant*" OR "stateless person*" OR "climate refugee*" OR "internally displaced person*" OR IDPs OR "economic migrant*" OR "temporary migrant*" OR "human trafficking victim*" OR "unaccompanied minor*" OR "migrant sex worker*" OR "female migrant*" OR "male migrant*" OR "migrant women" OR "migrant men" OR "LGBTQI+ migrant*" OR "sexual minority migrant*" OR "elderly migrant*" OR "aging migrant*" OR "migrant families" OR LEP OR "limited english proficiency" OR "newly arrived migrant*" OR "cross-border migrant*" OR expatriate* OR "seasonal migrant*" OR "guest worker*" OR "migrant healthcare worker*")

AND

TITLE-ABS-KEY("sexual health" OR "reproductive health" OR "family planning" OR contraception OR "induced abortion" OR "sexually transmitted disease*" OR "HIV infection*" OR AIDS OR "gender-based violence" OR "sexual dysfunction*" OR "sexual behavior" OR "sex education" OR "sexual and gender minorities" OR "intimate partner violence" OR "trauma-informed care" OR "unintended pregnancy" OR "postabortion care" OR "emergency contraception" OR "sexual rights" OR "reproductive rights" OR "sexual arousal" OR libido OR orgasm OR "sexual pain" OR "sexual satisfaction" OR intimacy OR sexuality OR "sexual violence" OR "sexual abuse" OR rape OR "forced marriage" OR "human trafficking" OR "female genital mutilation" OR "sexual exploitation" OR "sexual harassment" OR "survivors of sexual violence" OR "reproductive coercion" OR "sexual consent" OR "sexual autonomy" OR "sexual health education" OR "sexual and reproductive rights" OR "sexual well-being" OR "stigma and sexuality" OR "pleasure-centered sexual health" OR "migrant sexual health" OR "refugee reproductive health" OR "LGBTQ+ sexual health" OR "culturally sensitive sexual health services" OR "sexual dysfunction treatment" OR "mental health and sexual health")

AND

TITLE-ABS-KEY("health policy" OR "public policy" OR guideline* OR legislation OR "medical law" OR framework OR strategy OR "SRH policy" OR "national strategy" OR policy OR policies OR law*)

**Web of science**

**Date of Search:** 22 April 2025 **Search Filters Applied:** 2015–2025

**Number of Results:** 375

TS=("transients and migrants" OR refugees OR "refugee camps" OR "ethnic groups" OR "culturally and linguistically diverse" OR CALD OR "non-english speaking" OR "migrant worker*" OR "international student*" OR "labor migrant*" OR "minority group*" OR "asylum seeker*" OR "displaced people" OR immigrant* OR "undocumented migrant*" OR "illegal immigrant*" OR "stateless person*" OR "climate refugee*" OR "internally displaced person*" OR IDPs OR "economic migrant*" OR "temporary migrant*" OR "human trafficking victim*" OR "unaccompanied minor*" OR "migrant sex worker*" OR "female migrant*" OR "male migrant*" OR "migrant women" OR "migrant men" OR "LGBTQI+ migrant*" OR "sexual minority migrant*" OR "elderly migrant*" OR "aging migrant*" OR "migrant families" OR LEP OR "limited english proficiency" OR "newly arrived migrant*" OR "cross-border migrant*" OR expatriate* OR "seasonal migrant*" OR "guest worker*" OR "migrant healthcare worker*")

AND

TS=("sexual health" OR "reproductive health" OR "family planning" OR contraception OR "induced abortion" OR "sexually transmitted disease*" OR "HIV infection*" OR AIDS OR "gender-based violence" OR "sexual dysfunction*" OR "sexual behavior" OR "sex education" OR "sexual and gender minorities" OR "intimate partner violence" OR "trauma-informed care" OR "unintended pregnancy" OR "postabortion care" OR "emergency contraception" OR "sexual rights" OR "reproductive rights" OR "sexual arousal" OR libido OR orgasm OR "sexual pain" OR "sexual satisfaction" OR intimacy OR sexuality OR "sexual violence" OR "sexual abuse" OR rape OR "forced marriage" OR "human trafficking" OR "female genital mutilation" OR "sexual exploitation" OR "sexual harassment" OR "survivors of sexual violence" OR "reproductive coercion" OR "sexual consent" OR "sexual autonomy" OR "sexual health education" OR "sexual and reproductive rights" OR "sexual well-being" OR "stigma and sexuality" OR "pleasure-centered sexual health" OR "migrant sexual health" OR "refugee reproductive health" OR "LGBTQ+ sexual health" OR "culturally sensitive sexual health services" OR "sexual dysfunction treatment" OR "mental health and sexual health")

AND

TS=("health policy" OR "public policy" OR guideline* OR legislation* OR "medical law" OR framework* OR strategy* OR "SRH policy" OR "national strategy" OR policy* OR law*)

[**Public Health Database**](https://www.proquest.com/publichealth?accountid=8203)

**Date of Search:** 22 April 2025 **Search Filters Applied:** 2015–2025

**Number of Results:** 27, 305

TI,AB("transients and migrants" OR refugees OR "refugee camps" OR "ethnic groups" OR "culturally and linguistically diverse" OR CALD OR "non-english speaking" OR "migrant worker*" OR "international student*" OR "labor migrant*" OR "minority group*" OR "asylum seeker*" OR "displaced people" OR immigrant* OR "undocumented migrant*" OR "illegal immigrant*" OR "stateless person*" OR "climate refugee*" OR "internally displaced person*" OR IDPs OR "economic migrant*" OR "temporary migrant*" OR "human trafficking victim*" OR "unaccompanied minor*" OR "migrant sex worker*" OR "female migrant*" OR "male migrant*" OR "migrant women" OR "migrant men" OR "LGBTQI+ migrant*" OR "sexual minority migrant*" OR "elderly migrant*" OR "aging migrant*" OR "migrant families" OR LEP OR "limited english proficiency" OR "newly arrived migrant*" OR "cross-border migrant*" OR expatriate* OR "seasonal migrant*" OR "guest worker*" OR "migrant healthcare worker*")

AND

TI,AB("sexual health" OR "reproductive health" OR "family planning" OR contraception OR "induced abortion" OR "sexually transmitted disease*" OR "HIV infection*" OR AIDS OR "gender-based violence" OR "sexual dysfunction*" OR "sexual behavior" OR "sex education" OR "sexual and gender minorities" OR "intimate partner violence" OR "trauma-informed care" OR "unintended pregnancy" OR "postabortion care" OR "emergency contraception" OR "sexual rights" OR "reproductive rights" OR "sexual arousal" OR libido OR orgasm OR "sexual pain" OR "sexual satisfaction" OR intimacy OR sexuality OR "sexual violence" OR "sexual abuse" OR rape OR "forced marriage" OR "human trafficking" OR "female genital mutilation" OR "sexual exploitation" OR "sexual harassment" OR "survivors of sexual violence" OR "reproductive coercion" OR "sexual consent" OR "sexual autonomy" OR "sexual health education" OR "sexual and reproductive rights" OR "sexual well-being" OR "stigma and sexuality" OR "pleasure-centered sexual health" OR "migrant sexual health" OR "refugee reproductive health" OR "LGBTQ+ sexual health" OR "culturally sensitive sexual health services" OR "sexual dysfunction treatment" OR "mental health and sexual health")

AND

TI,AB("health policy" OR "public policy" OR guideline* OR legislation OR "medical law" OR framework OR strategy OR "SRH policy" OR "national strategy" OR policy OR policies OR law*)

AND

pd(20150101-20250414)

**CINAHL**

**Date of Search:** 22 April 2025 **Search Filters Applied:** 2015–2025

**Number of Results:** 356

(MH "Transients and Migrants+" OR MH "Refugees+" OR MH "Refugee Camps" OR MH "Ethnic Groups+")

OR

TI("culturally and linguistically diverse" OR CALD OR "non-english speaking" OR "migrant worker*" OR "international student*" OR "labor migrant*" OR "minority group*" OR "asylum seeker*" OR "displaced people" OR immigrant* OR "undocumented migrant*" OR "illegal immigrant*" OR "stateless person*" OR "climate refugee*" OR "internally displaced person*" OR IDPs OR "economic migrant*" OR "temporary migrant*" OR "human trafficking victim*" OR "unaccompanied minor*" OR "migrant sex worker*" OR "female migrant*" OR "male migrant*" OR "migrant women" OR "migrant men" OR "LGBTQI+ migrant*" OR "sexual minority migrant*" OR "elderly migrant*" OR "aging migrant*" OR "migrant families" OR LEP OR "limited english proficiency" OR "newly arrived migrant*" OR "cross-border migrant*" OR expatriate* OR "seasonal migrant*" OR "guest worker*" OR "migrant healthcare worker*") OR AB("culturally and linguistically diverse" OR CALD OR "non-english speaking" OR "migrant worker*" OR "international student*" OR "labor migrant*" OR "minority group*" OR "asylum seeker*" OR "displaced people" OR immigrant* OR "undocumented migrant*" OR "illegal immigrant*" OR "stateless person*" OR "climate refugee*" OR "internally displaced person*" OR IDPs OR "economic migrant*" OR "temporary migrant*" OR "human trafficking victim*" OR "unaccompanied minor*" OR "migrant sex worker*" OR "female migrant*" OR "male migrant*" OR "migrant women" OR "migrant men" OR "LGBTQI+ migrant*" OR "sexual minority migrant*" OR "elderly migrant*" OR "aging migrant*" OR "migrant families" OR LEP OR "limited english proficiency" OR "newly arrived migrant*" OR "cross-border migrant*" OR expatriate* OR "seasonal migrant*" OR "guest worker*" OR "migrant healthcare worker*")

AND

(MH "Sexual Health+" OR MH "Reproductive Health+" OR MH "Family Planning Services+" OR MH "Contraception+" OR MH "Abortion, Induced+" OR MH "Sexually Transmitted Diseases+" OR MH "HIV Infections+" OR MH "Acquired Immunodeficiency Syndrome" OR MH "Gender-Based Violence" OR MH "Sexual Dysfunctions, Psychological" OR MH "Sexual Behavior" OR MH "Sex Education" OR MH "Sexual and Gender Minorities" OR MH "Intimate Partner Violence" OR MH "Trauma-Informed Care" OR MH "Unintended Pregnancy" OR MH "Postabortion Care" OR MH "Emergency Contraception" OR MH "Sexual Rights" OR MH "Reproductive Rights") OR TI("sexual arousal" OR libido OR orgasm OR "sexual pain" OR "sexual satisfaction" OR intimacy OR sexuality OR "sexual violence" OR "sexual abuse" OR rape OR "forced marriage" OR "human trafficking" OR "female genital mutilation" OR "sexual exploitation" OR "sexual harassment" OR "survivors of sexual violence" OR "reproductive coercion" OR "sexual consent" OR "sexual autonomy" OR "sexual health education" OR "sexual and reproductive rights" OR "sexual well-being" OR "stigma and sexuality" OR "pleasure-centered sexual health" OR "migrant sexual health" OR "refugee reproductive health" OR "LGBTQ+ sexual health" OR "culturally sensitive sexual health services" OR "sexual dysfunction treatment" OR "mental health and sexual health") OR AB("sexual arousal" OR libido OR orgasm OR "sexual pain" OR "sexual satisfaction" OR intimacy OR sexuality OR "sexual violence" OR "sexual abuse" OR rape OR "forced marriage" OR "human trafficking" OR "female genital mutilation" OR "sexual exploitation" OR "sexual harassment" OR "survivors of sexual violence" OR "reproductive coercion" OR "sexual consent" OR "sexual autonomy" OR "sexual health education" OR "sexual and reproductive rights" OR "sexual well-being" OR "stigma and sexuality" OR "pleasure-centered sexual health" OR "migrant sexual health" OR "refugee reproductive health" OR "LGBTQ+ sexual health" OR "culturally sensitive sexual health services" OR "sexual dysfunction treatment" OR "mental health and sexual health")

AND

(MH "Health Policy+" OR MH "Public Policy" OR MH "Legislation, Medical") OR TI("guideline*" OR policy OR policies OR framework OR "law*" OR strategy OR "SRH policy" OR "national strategy")

OR AB("guideline*" OR policy OR policies OR framework OR "law*" OR strategy OR "SRH policy" OR "national strategy")
