# Supplementary material for: Sexual and reproductive health policies for migrant, immigrant and refugee populations in select high-income countries: a policy analysis protocol
Source: BMJ Open. 2025 Dec 10;15(12):e107994. doi: 10.1136/bmjopen-2025-107994 (PMC12699594; doi:10.1136/bmjopen-2025-107994)
Supplement: Supplementary file 3 [file bmjopen-2025-107994.reviewer_comments.pdf]

PEER REVIEW HISTORY

BMJ Open publishes all reviews undertaken for accepted manuscripts. Reviewers are asked to complete a checklist review form and are provided with free text boxes to elaborate on their assessment. These free text comments are reproduced below.

ARTICLE DETAILS

Title (Provisional)

Sexual and Reproductive Health Policies for Migrant, Immigrant, and Refugee Populations in Select High-Income Countries: A Policy Analysis Protocol

Authors

Mirzaei Damabi, Negin; Castleton, Patience; Mengesha, Zelalem; Munn, Z; Begum, Mumtaz; Avery, Jodie; Meherali, Salima; Lassi, Zohra

---

VERSION 1 - REVIEW

**Reviewer**	**1**
**Name**	**Erausquin, Jennifer Toller**
**Affiliation**	**UNC Greensboro School of Health and Human Sciences, Public Health Education**
**Date**	**08-Sep-2025**
**COI**	**None**

Bmjopen-2025-107994: "Policy Analysis Protocol: Access to Sexual and Reproductive Health for Migrant and Refugee Populations"

This is a protocol for a study that aims to analyze national-level sexual and reproductive health (SRH) policies across high income countries with high proportion of migrants and "strong migrant integration frameworks" (n=13). The authors plan to use a database search of peer-reviewed literature (and then review of the full text documents) plus a hand search of reports, etc. from key agencies to identify national and international sexual and reproductive health policies for migrants and refugees that apply to these 13 countries.

The language capacity of the authors for policy analysis does not seem to be mentioned, and that would be of key importance. Some of the document types the authors use for policy analysis are not clearly national or international policy (e.g., case reports and roundtable recommendations). It is also somewhat unclear why 13 high income countries from North America, Europe and Oceania are all included, given very different migration streams and policy contexts. Despite these concerns, with revisions this is likely to be a useful protocol, resulting in a publishable manuscript with implications for migrant and refugee SRH.

1. Suggested title: "Policy Analysis Protocol for National and International Sexual and Reproductive Health Policies for Migrant, Immigrant, and Refugee Populations in Select High-Income Countries."

2. Though mentioned in the abstract, it needs to be clear in the title and background that this study centers only on high-income countries. It is worth mentioning in the limitations that no countries from Asia, Africa, Central or South America, or the Caribbean are included.

3. It is not at all clear why the abstract mentions that "key gaps remain in addressing men's sexual health and women's sexual function." This sounds like results, which should not be included in a protocol paper. This same piece of information is used on p. 3 (background section), suggesting that the authors know what their proposed policy analysis will find—and the cited study is the same group of authors. Make clear how and why the proposed policy analysis is both new and unique research.

4. It is not entirely clear why, if the purpose of this analysis is to "strengthen Australia's SRH policy landscape," 12 other countries are included. Explain what purpose these other countries' policies serve.

5. The authors do a good job of defining sexual and reproductive health but also need to clearly define "migrant and refugee." How much time post-arrival in the country does a migrant become simply an immigrant? In addition, access to health care including SRH may vary greatly based on whether people have legal residency status in a country and whether they speak the language of that country. (This is mentioned on p. 3 but worth further consideration.)

6. Research questions: bullet point 2: The authors need to clarify that this question about whether the policy attempts to meet the needs of migrant and refugee populations, not whether those needs are actually met.

7. Additional justification is needed about why only countries with favorable policies (MIPEX score of 70 or higher) are included. There is vague mention of "contextual relevance" but the criteria for this other than high-income status is unclear.

8. What languages of results are your research team able to handle? The 13 countries have at least 8 languages in which policy documents would be written.

9. Table 3: Document Typology: It is unclear how technical reports, case studies, and roundtable recommendations count as national or international policy. A clearer definition of national / international policy is needed.

10. Of critical importance for a scoping review protocol: additional detail is needed for the description of methods for searching the grey literature. You may want to review this libguide (https://guides.library.unisa.edu.au/GreyLiterature/) and clearly explain that you plan to use both web-based grey literature searches (detail the search engines and keywords) and a search within the key agencies listed on p. 9. The remainder of the methods and analytic plan are described in clear detail.

11. It is not clear why two manuscripts will be written, one for policies for men and one for policies for women.

VERSION 1 - AUTHOR RESPONSE

Reviewer 1 comments:

1. The language capacity of the authors for policy analysis does not seem to be mentioned, and that would be of key importance. Some of the document types the authors use for policy analysis are not clearly national or international policy (e.g., case reports and roundtable recommendations). It is also somewhat unclear why 13 high income countries from North America, Europe and Oceania are all included, given very different migration streams and policy contexts. Despite these concerns, with revisions this is likely to be a useful protocol, resulting in a publishable manuscript with implications for migrant and refugee SRH.

Response:
Thank you very much for this thoughtful feedback. After conducting our initial search, we realised that almost all national SRH policies from non-English-speaking high-income countries were published exclusively in their native languages. Given the complexity of these documents, and our team's lack of capacity to translate and analyse them with adequate rigour, we decided to limit inclusion to English-speaking countries. We have clarified this language capacity constraint in the revised manuscript. Lines 54Regarding the use of the Migrant Integration Policy Index (MIPEX), we acknowledge the need to explain our criteria more clearly. MIPEX classifies countries into categories ranging from "Critical" (0–20), "Slightly Unfavourable" (21–40), "Halfway Favourable" (41–59), "Slightly Favourable" (60–79), through to "Favourable" (80–100). In the revised protocol we specify that we included countries scoring in the two higher brackets ("Slightly Favourable" and "Favourable"), but restricted this to English-speaking high-income countries for reasons of feasibility. Finally, we have revised and updated Table 2 to clarify the final list of included countries: Australia, Canada, Ireland, New Zealand, the United Kingdom, and the United States. (Lines 155-161) We also revised and clarified the types of documents eligible for inclusion in Table 4.

2. Suggested title: "Policy Analysis Protocol for National and International Sexual and Reproductive Health Policies for Migrant, Immigrant, and Refugee Populations in Select High-Income Countries."

Response:
The title has been revised, thank you for the suggestion.

3. Though mentioned in the abstract, it needs to be clear in the title and background that this study centers only on high-income countries. It is worth mentioning in the limitations that no countries from Asia, Africa, Central or South America, or the Caribbean are included.

Response:
Thank you for this important comment, We have added this point to the limitations of the study: "This study centres on only high-income countries, therefore no countries from Asia, Africa, Central or South America or the Caribbean are included, which may limit the relevance of outcomes to a global audience. " (Line 49-50)

4. It is not at all clear why the abstract mentions that "key gaps remain in addressing men's sexual health and women's sexual function." This sounds like results, which should not be included in a

protocol paper. This same piece of information is used on p. 3 (background section), suggesting that the authors know what their proposed policy analysis will find—and the cited study is the same group of authors. Make clear how and why the proposed policy analysis is both new and unique research.

Response:
Thank you for this helpful observation. We acknowledge that the phrasing in the abstract and background may have unintentionally implied preliminary results rather than highlighting existing evidence gaps. Our intention was not to pre-empt findings, but to emphasise why this policy analysis is needed. To clarify, we revised the abstract and background to frame men's sexual health and women's sexual function as well-documented gaps in the global SRH literature and policy landscape, rather than as findings of the current protocol. (Line 24)

It is not entirely clear why, if the purpose of this analysis is to "strengthen Australia's SRH policy landscape," 12 other countries are included. Explain what purpose these other countries' policies serve.

Response:
We appreciate this important point. Our ultimate aim is indeed to generate recommendations that strengthen Australia's SRH policy landscape, as this is where the research team is based and where the findings can have the most immediate impact. However, we intentionally included 12 other high-income countries that meet the criteria of having large migrant populations and favourable MIPEX health scores because they serve as critical comparators and benchmarks. By examining how other countries with similar demographic pressures and policy maturity address SRH for migrants and refugees, we can identify transferable lessons, innovative practices, and gaps that Australia can learn from. This comparative perspective also ensures the analysis is not only locally relevant but also contributes to the global policy discourse on migrant and refugee SRH. (Line 135 - 138 , Line 105 - 109)

5. The authors do a good job of defining sexual and reproductive health but also need to clearly define "migrant and refugee." How much time post-arrival in the country does a migrant become simply an immigrant? In addition, access to health care including SRH may vary greatly based on whether people have legal residency status in a country and whether they speak the language of that country. (This is mentioned on p. 3 but worth further consideration.)

Response:
Thank you for highlighting this important point. We agree that the definitions of "migrant" and "refugee" require greater clarity. To address this, we have added a new Table (Table 1) in the Methods, Population subsection that provides clear definitions of migrant, immigrant, refugee, asylum seeker, and undocumented migrant, drawing on authoritative sources. (Line 139)

6. Research questions: bullet point 2: The authors need to clarify that this question about whether the policy attempts to meet the needs of migrant and refugee populations, not whether those needs are actually met.

Response:
Thank you for this important point. We have now changed the wording of this dot point to read:
"To what extent do these SRH policies attempt to meet the needs of migrant and refugee populations, including undocumented migrants and asylum seekers?" (Line 102-103)

7. Additional justification is needed about why only countries with favorable policies (MIPEX score of 70 or higher) are included. There is vague mention of "contextual relevance" but the criteria for this other than high-income status is unclear.

Response:
Thank you for raising this important point. We have now clarified our rationale for selecting only countries with Slightly Favourable (MIPEX score 60–79) and Favourable (MIPEX score 80–100) health policy environments. We chose this threshold because our objective is to benchmark Australia's policies against other high-income settings with established or emerging strengths in migrant integration, rather than contexts where systemic barriers remain predominant. Including countries with lower MIPEX scores (≤59) would have highlighted deficits that are already well-documented, but would have provided limited transferable insights for informing policy development in Australia.
With respect to "contextual relevance," we have now specified that this refers to:
1. Health system comparability – all included countries are high-income with broadly similar institutional capacity to deliver comprehensive SRH care.
2. Migrant demographic significance – we restricted inclusion to countries where migrants comprise at least 10% of the population, ensuring that SRH policies are developed in contexts where migration meaningfully shapes health system demand.
3. Language feasibility – as noted, inclusion was further limited to English-speaking high-income countries, given our team's capacity to conduct detailed policy analysis only in English.

These clarifications have been added to the revised methods section, alongside an updated Table 2 that transparently lists the final set of countries (Australia, Canada, Ireland, New Zealand, the United Kingdom, and the United States). (Line 155-161)
We believe this added detail more clearly justifies the choice of MIPEX cut-off and the criteria for contextual relevance.

8. What languages of results are your research team able to handle? The 13 countries have at least 8 languages in which policy documents would be written. Thank you for this valuable observation. We agree that language capacity is a critical consideration in policy analysis. Initially, our inclusion criteria identified 13 high-income countries that met the MIPEX and migrant population thresholds; however, these countries collectively represent at least eight different policy languages. After piloting our search, we recognised that many national SRH policies from non-English-speaking countries were available only in their native languages. Given our team's limited budget and lack of specialist translation capacity, we were not able to rigorously analyse policies in multiple languages.

Response:
We have therefore revised our inclusion criteria to restrict the review to English-speaking high-income countries (Australia, Canada, Ireland, New Zealand, the United Kingdom, and the United States). This revision is now explicitly detailed in the Eligibility Criteria (line 161), and we have noted it as a limitation in the Strengths and Limitations section (lines 54–55):
"Due to the limited budget, authors only had the capacity to include documents published in English-language, thus further limiting the included countries to those whose primary language is English." We believe this clarification strengthens the transparency of our protocol and sets appropriate boundaries for the scope of analysis.

9. Table 3: Document Typology: It is unclear how technical reports, case studies, and roundtable recommendations count as national or international policy. A clearer definition of national / international policy is needed.
Response:
Thank you for this comment – we have now reduced our list of documents to include in the review as, upon further investigation we agree with your comment that case studies do not fit in as national/international policy. The rest of our included documents are listed in table 3. (Line 204)

10. Of critical importance for a scoping review protocol: additional detail is needed for the description of methods for searching the grey literature. You may want to review this libguide (https://guides.library.unisa.edu.au/GreyLiterature/) and clearly explain that you plan to use both web-based grey literature searches (detail the search engines and keywords) and a search within the key agencies listed on p. 9. The remainder of the methods and analytic plan are described in clear detail.

Response:
Changes to the methods section that details grey literature searches have been made to provide further description of methods for searching grey literature. Additionally, lines 228-231 have been updated to include more in-depth information:
"Web-based grey literature searches and searches within the above-mentioned key agencies will be conducted by hand using a consolidated, structured search strategy that follows the same structure as that of the one developed using MeSH terms. A more refined, simpler keyword search of grey literature will ensure that all relevant articles are screened for eligibility. Keywords such as "Migrant" OR "Refugee" OR "Asylum Seeker" OR "Culturally and Linguistically Diverse", and "Sexual Health" OR "Reproductive Health" OR "Family Planning" OR "HIV Treatment", and "Policy" OR "Policies" OR "Legislation" OR "Framework" OR "Guidelines" will be combined in multiple ways for simple 3–5-word searches of the grey literature. This will ensure that all relevant documents are captured and assessed for their relevance. "

11. It is not clear why two manuscripts will be written, one for policies for men and one for policies for women. Thank you for this point.

Response:
We have now added to the ethics and dissemination section of our manuscript to clearly state why this was decided:
"As this study involves available policy documents and does not involve human subjects, ethical approval is not required. The WHO Global Strategy for Women's, Children's and Adolescents' Health (2016-2030)and the Australian National Men's Health Strategy (2020-2030) similarly highlight the importance of sex-disaggregation in data and research, enabling the development of gender-specific intervention and policy approaches to ensure that no groups are overlooked and gender specific barriers are addressed. Therefore, two final policy review manuscripts, one focused specifically on SRH policies for women and the other on men, will be prepared following completion of the analysis, ensuring that gender inequalities in SRH policy are adequately addressed in the review. " (Line 301-306)

VERSION 2 - REVIEW

Reviewer 1

Name Erausquin, Jennifer Toller

Affiliation UNC Greensboro School of Health and Human Sciences, Public Health Education

Date 23-Oct-2025

COI

Bmjopen-2025-107994: "Policy Analysis Protocol: Access to Sexual and Reproductive Health for Migrant and Refugee Populations"

The authors have made important changes that improve the clarity and rigor of this protocol. The remaining concerns to be addressed are:

(1) the new final bullet of the Strengths and Limitations of this Study section: I suggest removing "due to the limited budget" and changing the tense to either present or future, not the past ("had the capacity").

(2) p. 4 Methods, additional text added: the work of the review cannot be conducted prior to the acceptance of the protocol. Please adjust the dates.

(3) Point 11 of prior review comments: You are correct that disaggregating data is an important practice. It is still not clear why two separate manuscripts are planned, using the exact same methods, search strategy, data extraction tool, etc.

VERSION 2 - AUTHOR RESPONSE

Reviewer One comments	Author Response
(1) the new final bullet of the Strengths and Limitations of this Study section: I suggest removing "due to the limited budget" and changing the tense to either present or future, not the past ("had the capacity").	Thank you for this important point, we have now changed the dot point as follows:<br>"Authors only have the capacity to include documents published in English-language, thus further limiting the included countries to those whose primary language is English. "<br>Line 53-54
(2) p. 4 Methods, additional text added: the work of the review cannot be conducted prior to the acceptance of the protocol. Please adjust the dates.	Thank you for picking this up! We have now adjusted our dates to:<br>November 2025 and February 2026<br>Line 133-134
(3) Point 11 of prior review comments: You are correct that disaggregating data is an important practice. It is still not clear why two separate manuscripts are planned, using the exact same methods, search strategy, data extraction tool, etc.	Thank you for this comment, we have made some edits to this section to ensure we are clearly stating why the two separate manuscripts are needed.<br>"Although the same methodology, search strategy and data extraction tool will be applied, the complexity and distinct nature of gendered policy issues warrant separate in-depth reporting and analysis. Additionally, to provide an in-depth cross-domain analysis of policy covering all WHO-SRH domains two papers are needed. Therefore, two complementary policy review manuscripts, one focused specifically on SRH policies for women and the other on men, will be prepared following completion of the

	analysis, ensuring that gender inequalities in SRH policy are adequately addressed in the review. Keeping the methodology the same within the two manuscripts will be important in allowing direct comparison between women's and men's SRH policies, ensuring both methodological rigour and the ability to cross-check findings for gender-based similarities and differences. This will allow for nuanced interpretation of gender-specific policy contexts, priorities and gaps, ensuring that the unique health needs and systemic inequalities affecting each group are adequately addressed."<br>Line 332-341
